# Visual task-related functional and structural magnetic resonance imaging for the objective quantitation of visual function in patients with advanced retinitis pigmentosa

**DOI:** 10.3389/fnagi.2022.825204

**Published:** 2022-07-22

**Authors:** Hao Wang, Wangbin Ouyang, Yong Liu, Minfang Zhang, He Zhao, Jian Wang, Zhengqin Yin

**Affiliations:** ^1^Southwest Hospital/Southwest Eye Hospital, Army Medical University, Chongqing, China; ^2^Key Lab of Visual Damage and Regeneration and Restoration of Chongqing, Chongqing, China; ^3^Department of Radiology, Southwest Hospital, Army Medical University, Chongqing, China

**Keywords:** advanced retinitis pigmentosa, functional magnetic resonance imaging, visual task, response intensity, functional connectivity, gray matter volume

## Abstract

**Purpose:**

The objective quantitation of visual function in patients with advanced retinitis pigmentosa (RP) presents a difficult challenge due to the weak visual function of these patients. This study utilized magnetic resonance imaging (MRI) to assess the function and structure of the visual cortex (VC) in patients with RP and quantitatively categorize them.

**Materials and Methods:**

Twenty-three patients with RP and ten healthy controls (HCs) were enrolled for MRI examinations. The patients were divided into form perception (FP) and no form perception (NFP) groups. Participants underwent structural MRI scans, and two visual task functional MRI scans were performed using stimuli, including white flash and black and white checkerboard patterns. Eight regions of interest (ROIs) were studied. In structural MRI, the gray matter volume (GMV) was compared in the ROIs. In the two visual tasks, the response intensity and functional connectivity (FC) of ROIs were also compared separately. Correlation analysis was performed to explore the correlations between the structural and functional parameters.

**Results:**

In the structural analysis, the GMV in Brodmann areas 17, 18, and 19 of the FP and NFP groups was significantly lower than that of HCs. Regarding the functional data, the response intensity in the VC of both the FP and NFP groups was significantly lower than that in HCs. The response in Brodmann areas 17, 18, and 19 obtained using the pattern stimulus was significantly lower in the NFP group than in the FP group. For the FC comparison, the FP and NFP groups exhibited significantly lower values in several pathways than the HCs, and FC in the ipsilateral V1–contralateral V1 pathway in the flash task was significantly lower in the NFP group than in the FP group. A positive correlation between response intensity and GMV was observed in Brodmann areas 17, 18, and 19 in both flash and pattern visual tasks.

**Conclusion:**

Magnetic resonance imaging was an effective tool to objectively and quantitatively evaluate the visual function of patients with advanced RP. Response intensity and FC were effective parameters to distinguish FP and NFP patients. A positive correlation between response intensity and GMV was observed in the VC.

## Introduction

Retinitis pigmentosa (RP) is a blinding hereditary retinal disease that leads to progressive visual impairment (Zobor and Zrenner, 2012). The global incidence rate is approximately 1/3000–1/4000 (Fahim, 2018). The loss of retinal photoreceptors is a primary symptom of retinal degeneration, and its clinical features include night blindness and tunnel vision; in the advanced stage of the disease, patients lose all or nearly all of their vision (Wang et al., 2019). These patients cannot be assessed using conventional visual field (VF) and visual electrophysiological examinations due to poor visual function and can only report visual acuity in terms of counting fingers (CF), hand movement (HM), light perception (LP), or no light perception (NLP). The examination results are subjective and are not objective or quantitative. However, the objective and quantitative examination of visual function in patients with advanced RP is a critical issue in RP therapy research because the therapeutic effects of most treatments must be evaluated. Therefore, objectively and quantitatively evaluating visual function in patients with advanced stages of RP has been a major challenge (Rachitskaya and Yuan, 2016; Dias et al., 2018).

Therapy for RP, including techniques such as gene therapy (Nuzbrokh et al., 2021; Wagner et al., 2021), stem cell therapy (Ahmed et al., 2021; Sharma and Jaganathan, 2021), and photogenetic therapy (Klapper et al., 2016; Liu et al., 2016), has been a central research focus in ophthalmology. At present, most of the therapeutic methods used in animal studies have shown the potential to increase visual function *in vivo*. The clinical trials performed in the next step also require objective and quantitative assessment methods to evaluate visual function; however, an effective examination that objectively and quantitatively discriminates form perception (FP) and no form perception (NFP) patients with advanced RP is unavailable. Therefore, a meaningful approach is to develop an examination method that objectively and quantitatively assesses the visual function of patients with advanced RP before and after treatment.

Functional magnetic resonance imaging (fMRI) is a non-invasive imaging technology that has been used to detect brain activation and functional connectivity (FC) (Culham et al., 1998; Rogers et al., 2007). The basic principle of fMRI entails measurements of changes in blood oxygen level-dependent (BOLD) signals during brain activation. fMRI has been widely used in studies of visual function and other brain functions for more than 20 years because of its superior ability to spatially localize sources of activity in the brain (Cox and Savoy, 2003; Logothetis, 2008). Although patients with advanced RP have a very weak visual function, they still receive some visual input, such as LP and HMs, or count figures. In our previous study, we found that the flash-evoked visual potential elicits a signal in the visual cortex (VC) of patients with advanced RP, even in patients with NLP, and fMRI showed the potential to assess vision-related brain function in blind patients with RP (Zhang et al., 2021). However, we still do not know whether fMRI is useful to differentiate various visual functional levels in patients with advanced RP. If fMRI was shown to be a feasible method for identifying differences in the brain function of FP and NFP patients, this technique would be valuable for evaluating the efficacy of RP therapy.

In this study, we observed the function and structure of visual brain areas in patients with advanced RP using magnetic resonance imaging (MRI). The areas evaluated included the left and right VC, superior parietal lobule (SPL), inferior temporal gyrus (ITG), and frontal eye fields (FEF), which are the main nodes in the dorsal and ventral streams (Nassi and Callaway, 2009; Rosa et al., 2009). The identification of visual task-related functions of key brain areas and the FC among the areas might help us assess the visual function of patients with advanced RP and develop a potential method to distinguish different visual functional levels in these patients.

## Materials and methods

### Study population

All patients with advanced RP were diagnosed by three senior ophthalmologists at the Southwest Hospital Eye Institute, Army Medical University, Chongqing, China. All subjects underwent routine clinical examinations, including best-corrected visual acuity (BCVA), an intraocular pressure (IOP) assessment, slit-lamp examination, color fundus photography (CFP), optical coherence tomography (OCT), fundus autofluorescence (FAF), fluorescein fundus angiography (FFA), VF analysis, and clinical visual electrophysiology, coupled with a review of each patient’s medical history of night blindness, family history, and other information.

**FIGURE 1:**
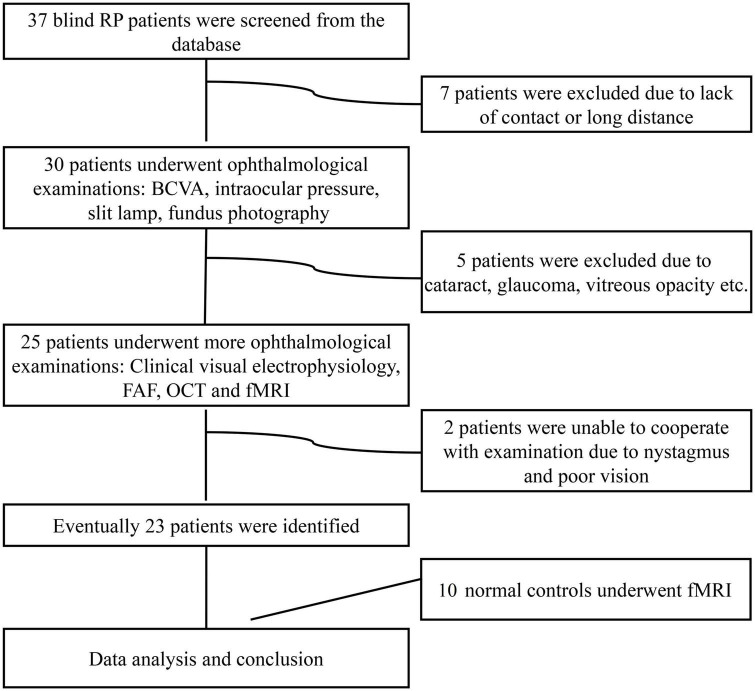
Flow diagram of the patient selection process. RP, retinitis pigmentosa; BCVA, best-corrected visual acuity; FAF, fundus autofluorescence; OCT, optical coherence tomography; fMRI, functional magnetic resonance imaging.

The study enrolled 23 subjects with RP from a pool of 37 patients with advanced RP (see Figure 1). The detailed clinical information for the subjects is shown in Table 1. All patients with RP had equal visual levels in both eyes (FP or NFP). FP indicates that the BCVA was at the level of HM detection or better, and NFP denotes patients whose BCVA was at the level of LP or worse. The inclusion criteria included male or female patients with RP aged 18–60 (inclusive) years, and the BCVA of both eyes was worse than 3/60 or the VF radius was less than 10 degrees (legal blindness) (Khoury et al., 2017). The exclusion criteria included the presence of any other ocular disorder, such as severe cataract, glaucoma, conjunctivitis, keratitis, scleritis, uveitis, endophthalmitis, retinal vascular occlusion, retinal detachment, macular hole, vitreous macular traction, or other relevant disorders; a history of intraocular surgery in the previous 6 months; and a history of stroke, coronary heart disease, or pregnancy.

**TABLE 1:** Detailed clinical characteristics of patients with RP.

Subject	Age (years)	Sex	Handedness	Onset of symptoms (years)	Disease duration (years)	Visual acuity (decimals)	
						RE	LE
RP001	34	M	R	19	15	HM	CF
RP002	54	M	R	41	13	HM	HM
RP003	45	F	R	25	20	1/20	3/100
RP004	57	F	R	40	17	LP	LP
RP005	55	M	R	42	13	NLP	NLP
RP006	18	M	R	10	8	1/100	1/100
RP007	30	M	R	22	8	HM	HM
RP008	51	F	R	26	25	HM	HM
RP009	57	M	R	27	30	HM	HM
RP010	26	F	R	7	19	1/20	1/20
RP011	20	M	R	2	18	HM	0.01
RP012	50	F	R	8	42	NLP	LP
RP013	42	M	R	20	22	LP	LP
RP014	59	M	R	9	50	LP	LP
RP015	34	M	R	8	26	LP	NLP
RP016	56	M	R	10	46	LP	LP
RP017	33	F	R	14	19	LP	LP
RP018	60	F	R	11	50	LP	LP
RP019	47	M	R	20	27	LP	LP
RP020	56	F	R	24	32	NLP	NLP
RP021	38	F	R	17	19	HM	HM
RP022	57	M	R	18	39	LP	LP
RP023	56	F	R	23	33	HM	HM

RP, retinitis pigmentosa; M, male; F, female; R, right; RE, right eye; LE, left eye; NLP, no light perception; LP, light perception; HM, hand movement; CF, counting finger; BCVA, best-corrected visual acuity.

### Magnetic resonance imaging data acquisition

All MR images were acquired at the Department of Radiology of the Southwest Hospital, Chongqing, China, using a 3.0 Tesla MR scanner (Trio Tim system; Siemens, Germany) equipped with an eight-channel phased-array head coil. Foam padding was used to limit head movement and reduce scanner noise.

T1-weighted structural images were acquired using a magnetization-prepared rapid gradient-echo imaging sequence with the following scan parameters: 1 mm × 1 mm × 1 mm voxel size, repetition time (TR) = 2530 ms, echo time (TE) = 2.34 ms, flip angle (FA) = 7°, matrix = 192 × 256, field of view (FOV) = 256 mm × 256 mm, slice thickness = 1 mm, slice gap = 0 mm, and 192 sagittal slices. The total scan time was approximately 20 min. All subjects were required to keep their eyes closed, keep their heads still, and remain awake during the scan.

We designed two visual tasks to evoke the LP function (a flash visual task) and FP function (a pattern visual task) of the subjects as a method to assess the key visual brain areas in the dorsal and ventral visual streams. Both visual tasks included eight repeated blocks, and each block lasted 1 min and was composed of two conditions: The first was the black condition, which entailed exposure to complete darkness lasting 30 s, and the second condition entailed 30 s of light exposure. Two types of visual stimulation were used: a white flash and a white-black reversing checkerboard (Supplementary Videos 1, 2).

For the task-related fMRI recording, the functional images consisted of echo-planar imaging (EPI) acquired with the following parameters: 3 mm × 3 mm × 3 mm voxel size, TR = 2 s, TE = 30 ms, FA = 90°, FOV = 192 mm × 192 mm, slices = 180, and imaging matrix = 64 × 64. The stimulus programs were designed by E-prime 2.0 (Psychology Software Tools, Inc., Pittsburgh, PA, United States). The stimulus flickering frequency was 8 Hz (Jaillon et al., 2007), and the visual tasks were presented monocularly with refraction correction by MRI-compatible goggles separately.

### Magnetic resonance imaging data processing

All visual fMRI data were pre-processed using the toolbox DPABI^1^ (Yan et al., 2016) based on Statistical Parametric Mapping 12^2^ and executed in MATLAB 2013b (MathWorks, Natick, MA, United States). All data were processed as follows: conversion of all raw images from DICOM format into NIfTI format; realignment of 180 volumes of the functional images to eliminate the effect of head motion; registration of T1-weighted structural images to the mean function images; and reslicing of the normalized data at a resolution of 3 mm × 3 mm × 3 mm. Smoothing [full width at half maximum (FWHM) = 8 mm] was used to reduce the noise effect, and the specific first-level module of SPM was used to convert the BOLD value to the con value for statistical analysis. The second-level analysis was performed using analysis of variance (ANOVA) in all groups. Peak-level topological false discovery rate (FDR) correction was used to correct for multiple comparisons. The BrainNet Viewer^3^ toolbox was selected to show the activation maps of the VC (Xia et al., 2013).

The structural data were pre-processed using the VBM8 toolbox^4^ in the SPM12 software package running in MATLAB (Farokhian et al., 2017). The image set of each subject was spatially normalized to the Montreal Neurological Institute (MNI) template using the high-dimensional DARTEL algorithm. The normalized images were segmented into gray matter (GM), white matter (WM), and cerebrospinal fluid (CSF). Then, the modulated GM images were smoothed with an 8-mm FWHM isotropic Gaussian kernel. Finally, the smoothed GM images were resampled to a 3 mm × 3 mm × 3 mm voxel size for the statistical analysis.

We defined eight regions of interest (ROIs) related to visual processing in the VC, SPL, ITG, and FEF in both hemispheres by anatomical location based on standard MRI structural coordinates in MNI space. The VC was defined as the sum of Brodmann areas 17, 18, and 19 in the anatomical MRI mask of the SPM toolbox xjView^5^ (Kawachi, 2017). The SPL and ITG were also defined using an xjView mask, and the FEF was defined as the area around the junction of the precentral gyrus and the middle frontal gyrus based on MNI coordinates from previous human fMRI research (Culham et al., 1998; Kawashima et al., 1998). An 8 mm × 8 mm × 8 mm area in each hemisphere was specified to be the mask for the FEF ROI.

The MRI data from the eight ROIs were collected for statistical analysis. The data collected included the response intensities (β-value) of the eight ROIs, which were analyzed using SPM 12, and FC correlation coefficients among the ROIs for the flash and pattern fMRI, which were analyzed using the REST toolkit.^6^ The gray matter volume (GMV) of the eight ROIs was determined from structural MRI using VBM8. The VC ROI was further divided into Brodmann areas 17, 18, and 19 to study the response intensity and structural data to achieve greater spatial precision. However, the VC was considered a single ROI for the purposes of the FC study because the interregional distances of Brodmann areas 17, 18, and 19 are relatively small.

### Statistical analyses

In visual task fMRI analysis, participants in the FP and NFP groups were compared with the participants in the control group separately. The ROIs were divided into ipsilateral ROIs of the stimulated eye and contralateral ROIs of the stimulated eye for statistical analysis. In structural MRI analysis, the participants in the FP and NFP groups were also compared with the participants in the control group separately, and the ROIs were divided by the left and right hemispheres of the subject. The response intensity (β-value) and FC value in flash and pattern fMRI and the GMV value from structural MRI were compared separately.

All statistical analyses were performed using SPSS software (version 25.0; SPSS, Inc., Chicago, IL, United States). Normality was verified first by performing the Shapiro–Wilk test. For data with a normal distribution, an independent-samples *t*-test or ANOVA was performed. *Post hoc* testing was performed using the least significant difference (LSD) method in SPSS, and statistical significance was set to *P* < 0.05. Bonferroni correction was used to correct the mistakes in multiple comparisons, and the adjusted *P*-value was set to *P_adj* < 0.05 in the preceding ANOVA. Spearman’s rank correlation analysis was conducted to explore the correlation between the function and structure parameters of key ROIs.

## Results

### Clinical details of patients with retinitis pigmentosa and control subjects

Twenty-three patients with RP (46 eyes), aged 18–60 years (median 45 years), and 10 control patients (20 eyes), aged 18–57 years (median 42 years), were recruited. According to their BCVA values, the patients with RP were categorized into a FP group and a NFP group. The FP group included 11 patients (six males, five females; age 18–57 years, median 39 years), and the NFP group included 12 patients (seven males, five females; aged 33–60 years, median 50.5 years). ANOVA was performed for the age of the three groups (*F* = 2.611, *P* = 0.090). The details of the patients with RP are shown in Table 1.

### The routine clinical examination of patients with retinitis pigmentosa and healthy controls

**FIGURE 2:**
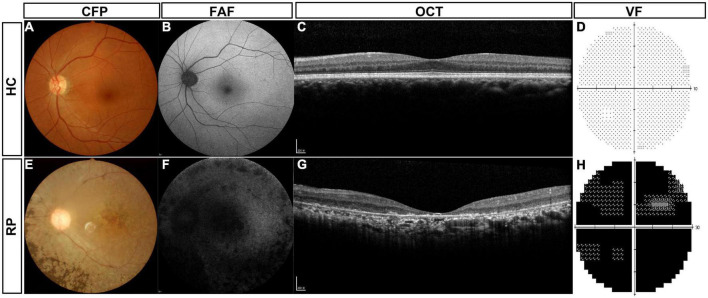
Routine clinical examinations of typical patients with RP and HC subjects. **(A)** CFP of HC subjects, **(B)** FAF of HC subjects, **(C)** macular OCT of HC subjects, **(D)** VF of HC subjects, **(E)** CFP of typical patients with RP, **(F)** FAF of typical patients with RP, **(G)** macular OCT of typical patients with RP, and **(H)** VF of typical patients with RP. HC, healthy control; RP, retinitis pigmentosa; CFP, color fundus photography; FAF, fundus autofluorescence; OCT, optical coherence tomography; VF, visual field.

The routine clinical examinations of typical patients with RP and normal control subjects are shown in Figure 2. Healthy controls (HCs) had a normal retinal structure and function (Figures 2A–D). In the CFP examination, patients with RP typically showed pigment degeneration (Figure 2E), and FFA showed that the patients with RP lost most of the retinal pigment epithelium (Figure 2F). Macular OCT showed the degeneration of photoreceptors (Figure 2G), and the patients with RP also lost most of the VF (Figure 2F).

### Comparison of the gray matter volume of vision-related regions of interest between patients with retinitis pigmentosa and healthy controls

**FIGURE 3:**
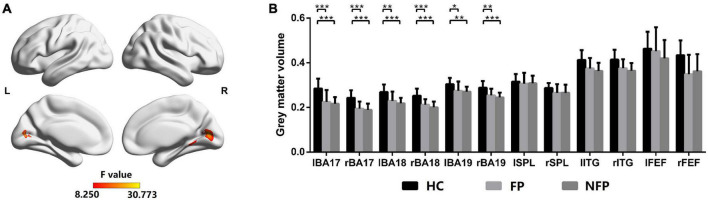
Comparison of GMVs of participants in the FP, NFP, and HC groups. **(A)** The significant differences in GMV across brain areas in participants in the three groups (*F*-values). **(B)** Comparison of GMVs in Brodmann areas 17, 18, and 19 and in the SPL, ITG, and FEF areas between participants in the three groups. HC, healthy control; FP, form perception; NFP, no form perception; lBA, left Brodmann area; rBA, right Brodmann area; lSPL, left superior parietal lobule; rSPL, right superior parietal lobule; lITG, left inferior temporal gyrus; rITG, right inferior temporal gyrus; lFEF, left frontal eye field; rFEF, right frontal eye field. ***P_adj < 0.001, **P_adj < 0.01, and *P_adj < 0.05.

The GMV of vision-related ROIs was the structural basis of visual function in patients with RP and HCs; therefore, the GMV of vision-related ROIs was compared first. Figure 3 shows the comparison of GMVs among the participants in the FP, NFP, and HC groups. The brain areas with significantly different GMVs are shown in Figure 3A (*F* = 8.250–30.773, *P_adj* < 0.05, FDR correction). The significantly different areas are colored yellow and red, and the VC showed obvious differences. The ROI GMV analysis and comparison are shown in Figure 3B. The VC ROI was also divided into Brodmann areas 17, 18, and 19 for a more precise comparison, and the GMV data were grouped by the left and right cerebral hemispheres for statistical analysis.

Analysis of variance was performed for GMV with Bonferroni correction in the left and right Brodmann areas 17, 18, and 19. In the left Brodmann area 17 (*F* = 8.872, *P_adj* = 0.012), right Brodmann area 17 (*F* = 12.450, *P_adj* = 0.001), left Brodmann area 18 (*F* = 7.609, *P_adj* = 0.028), right Brodmann area 18 (*F* = 13.590, *P_adj* < 0.001), left Brodmann area 19 (*F* = 6.411, *P_adj* = 0.048), and right Brodmann area 19 (*F* = 7.113, *P_adj* = 0.036), the GMV of participants in the FP and NFP groups was significantly lower than that of HCs, but no significant difference was noted in these ROIs between participants in the FP and NFP groups. No significant difference in GMV was observed between the three groups in the left and right SPL, ITG, and FEF areas. Therefore, the structural difference in patients with advanced RP was mainly located in the VC but not in higher vision-related brain regions, as the structural difference was not obvious in participants in the FP and NFP groups (Figure 3B).

### Comparison of the response intensity of vision-related regions of interest among patients with retinitis pigmentosa and healthy controls in flash and pattern visual tasks

**FIGURE 4:**
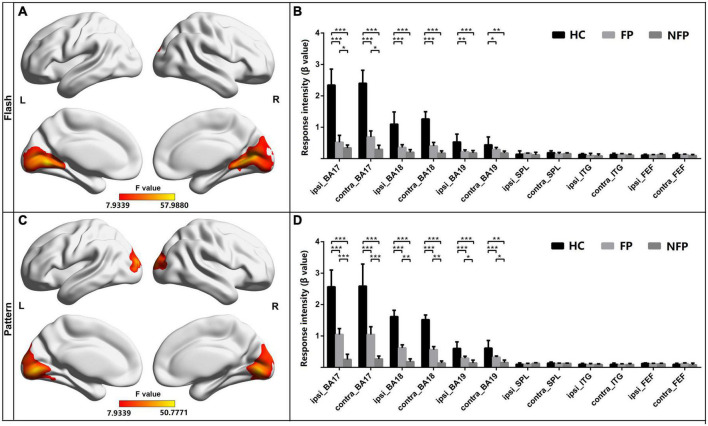
Comparison of the response intensity of participants in the FP, NFP, and HC groups in the flash and pattern visual tasks. **(A)** Significant differences (*F*-values) were observed in response intensity across brain areas for participants in the three groups in the flash visual task. **(B)** Comparison of the response intensity in Brodmann areas 17, 18, and 19 and in SPL, ITG, and FEF areas of participants in the three groups in the flash task. **(C)** Brain areas with significantly different response intensities in participants in the three groups in the pattern visual task. **(D)** Comparison of the intensity of the response in both Brodmann areas 17, 18, and 19 and in the SPL, ITG, and FEF areas to the pattern stimulus for participants in the three groups. HC, healthy control; FP, form perception; NFP, no form perception; ipsi_BA, ipsilateral Brodmann area; contral_BA, contralateral Brodmann area; ipsi_SPL, ipsilateral superior parietal lobule; contral_SPL, contralateral superior parietal lobule; ipsi_ITG, ipsilateral inferior temporal gyrus; contral_ITG, contralateral inferior temporal gyrus; ipsi_FEF, ipsilateral frontal eye field; contral_FEF, contralateral frontal eye field. ***P < 0.001, **P < 0.01, and *P < 0.05.

The neural function of visually related ROIs in the three groups was also compared. All patients with RP and HC subjects performed flash and pattern visual tasks. For participants in the FP, NFP, and HC groups, the brain areas showing significantly different activation in the flash visual task are depicted in Figure 4A (*F* = 7.934–57.988, *P_adj* < 0.05, FDR correction), and the brain areas showing significantly different activation in the pattern visual task are shown in Figure 4C (*F* = 7.934–50.777, *P_adj* < 0.05, FDR correction). The red and yellow brain areas show significant differences (*F*-value) in activation during the visual tasks. The VC, including Brodmann areas 17, 18, and 19, showed obvious differences in response to both flash and pattern stimuli.

For the ROI analysis in the flash visual task, the ROIs were grouped as those ipsilateral to the stimulated eye and those contralateral to the stimulated eye. ANOVA with Bonferroni correction was performed on the response intensity in the three groups. In Brodmann area 17 ipsilateral to the stimulated eye (*F* = 29.759, *P_adj* < 0.001) and Brodmann area 17 contralateral to the stimulated eye (*F* = 27.322, *P_adj* < 0.001), the response intensity measured in participants in the HC group was significantly higher than that of participants in the FP and NFP groups. In addition, in the two ROIs, the response intensity in participants in the FP group was significantly higher than that of participants in the NFP group. In Brodmann area 18 ipsilateral to the stimulated eye (*F* = 22.199, *P_adj* < 0.001), Brodmann area 18 contralateral to the stimulated eye (*F* = 23.245, *P_adj* < 0.001), Brodmann area 19 ipsilateral to the stimulated eye (*F* = 8.346, *P_adj* = 0.012), and Brodmann area 19 contralateral to the stimulated eye (*F* = 5.650, *P_adj* = 0.048), the response intensity detected in participants in the HC group was significantly higher than that of participants in the FP and NFP groups, but a significant difference was not observed in the four ROIs between participants in the FP and NFP groups. No significant difference in response intensity was observed in the other ROIs between the three groups, including the ipsilateral and contralateral SPL, ITG, and FEF areas, using ANOVA. Please see Figure 4B.

For the ROI analysis in the pattern visual task, the response intensity of ROIs in the three groups was also compared using the same approach. In Brodmann area 17 ipsilateral to the stimulated eye (*F* = 24.164, *P_adj* < 0.001), Brodmann area 17 contralateral to the stimulated eye (*F* = 29.115, *P_adj* < 0.001), Brodmann area 18 ipsilateral to the stimulated eye (*F* = 28.504, *P_adj* < 0.001), Brodmann area 18 contralateral to the stimulated eye (*F* = 33.497, *P_adj* < 0.001), Brodmann area 19 ipsilateral to the stimulated eye (*F* = 12.315, *P_adj* < 0.001), and Brodmann area 19 contralateral to the stimulated eye (*F* = 10.564, *P_adj* = 0.001), the response intensity of HCs was significantly higher than that of participants in the FP and NFP. Moreover, the response intensity in the six ROIs of participants in the FP group was significantly higher than that of participants in the NFP group. No response significant differences in intensity were observed in other ROIs, including the ipsilateral and contralateral SPL, ITG, and FEF areas, between the three groups (please see Figure 4D).

In the comparison of the neural function of vision-related ROIs, we found that the participants in the FP and NFP groups showed significant differences in neural function in the VC; moreover, the pattern visual task was a more effective visual task to differentiate patients in the FP and NFP groups. However, differences in neural function were not observed in higher vision-related brain regions.

### Comparison of the functional connectivity of vision-related regions of interest between patients with retinitis pigmentosa and healthy controls in flash and pattern visual tasks

**FIGURE 5:**
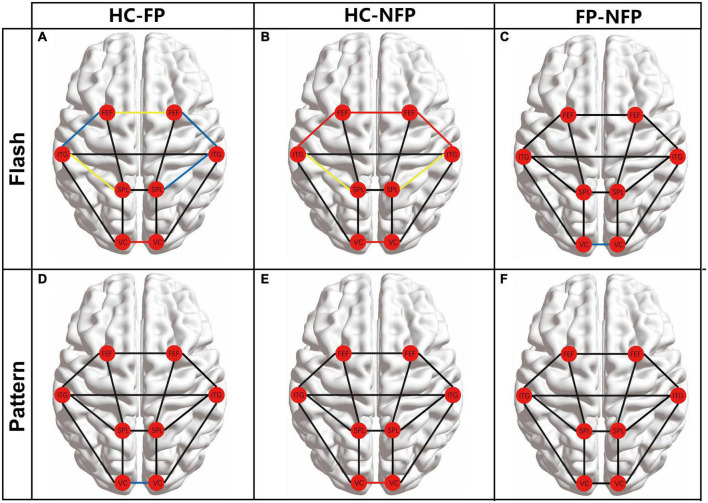
Comparison of the main FC between the eight ROIs among participants in the three groups. **(A)** Comparison of FC between participants from the HC and FP groups in the flash visual task. **(B)** FC comparison of FC between participants from the HC and NFP groups in the flash visual task. **(C)** Comparisons of FC between the FP and NFP groups in the flash visual task. **(D)** Comparison of FC between participants in the HC and FP groups in the pattern visual task. **(E)** Comparison of FC between participants in the HC and NFP groups in the pattern visual task. **(F)** Comparisons of FC between the FP and NFP groups in the pattern visual task. HC, healthy control; FP, form perception; NFP, no form perception; VC, visual cortex; SPL, superior parietal lobule; ITG, inferior temporal gyrus; FEF, frontal eye fields. Red line: *P* < 0.001, yellow line: *P* < 0.01, blue line: *P* < 0.05, and black line: *P* ≥ 0.05.

The FC among the eight ROIs was compared between participants in the three groups to assess the neural function of higher brain regions. The main FC correlation coefficient of the eight ROIs in flash and pattern tasks was compared using ANOVA with Bonferroni correction. The results of the *post hoc* multiple comparisons test are shown in Figure 5. The left cerebral hemisphere represented the hemisphere ipsilateral to the stimulated eye, and the right cerebral hemisphere represented the hemisphere contralateral to the stimulated eye. The red circles represent the locations of the ROIs, and the lines between the circles represent the FC between the pairs of ROIs. Black lines represent no significant difference in the ANOVA with Bonferroni correction, blue lines represent a significant difference with a *P_adj*-value less than 0.05 but greater than 0.01, yellow lines represent a significant difference with a *P_adj*-value less than 0.01 but greater than 0.001, and red lines represent a significant difference with a *P_adj*-value less than 0.001.

In the flash visual task, FC between some regions showed significant differences between participants in the three groups according to ANOVA with Bonferroni correction, including ipsi_V1–contra_V1 (*F* = 21.567, *P_adj* < 0.001), ipsi_SPL–ipsi_ITG (*F* = 7.390, *P_adj* = 0.022), contra_SPL–contra_ITG (*F* = 6.803, *P_adj* = 0.032), ipsi_ITG–ipsi_FEF (*F* = 8.195, *P_adj* = 0.013), contra_ITG–contra_FEF (*F* = 9.176, *P_adj* = 0.005), and ipsi_FEF–contra_FEF (*F* = 10.604, *P_adj* = 0.002). In these pathways, the FC of participants in both the FP and NFP groups was significantly lower than that of the HCs (Figures 5A,B). Moreover, the FC in the ipsi_V1–contra_V1 pathway of participants in the NFP group was significantly lower than that of participants in the FP group (Figure 5C). No significant difference in FC in other pathways was observed between the three groups.

In the pattern visual task, only ipsi_V1–contra_V1 FC showed a significant difference between participants in the three groups (*F* = 10.252, *P_adj* = 0.002). The values for participants in the FP group and NFP group were significantly lower than those for HCs (Figures 5D,E), but a significant difference in values was not observed between participants in the FP group and the NFP group (Figure 5F). No significant difference in FC was detected in other pathways between the three groups.

### Correlation analysis of the average response intensity in visual tasks and average gray matter volume in Brodmann areas 17, 18, and 19 of patients with retinitis pigmentosa and healthy controls

Both cerebral cortical function (response intensity in visual tasks) and structure (GMV) of the three groups were significantly different in Brodmann areas 17, 18, and 19. Therefore, we performed a correlation analysis of the response intensity in visual tasks and the GMV of related ROIs to study the relationship between the function and structure of ROIs. We averaged the response intensity of the corresponding ipsilateral and contralateral ROIs in the same visual task of the left and right eyes and averaged the GMV of the corresponding left and right ROIs to build the one-to-one relationship between function and structure in each ROI.

**FIGURE 6:**
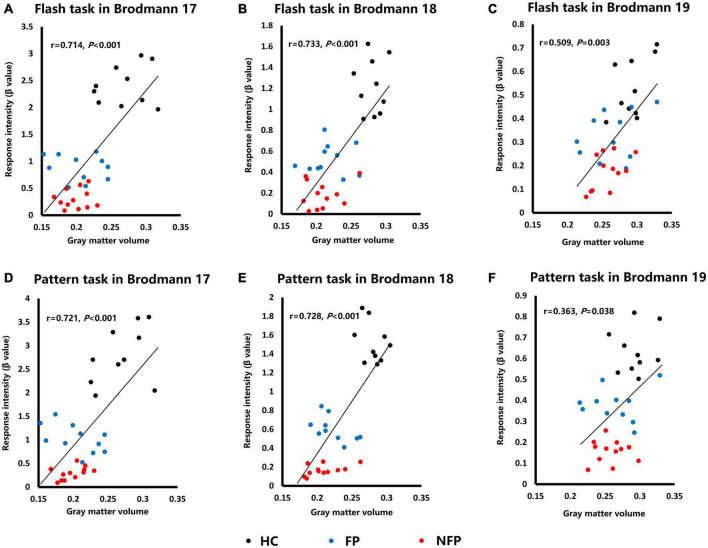
Plots of the correlation analysis of response intensity in visual tasks and GMVs of Brodmann areas 17, 18, and 19. **(A)** Correlation analysis of data obtained from Brodmann area 17 in response to the flash stimulus. **(B)** Correlation analysis of data obtained from Brodmann area 18 in response to the flash stimulus. **(C)** Correlation analysis of data obtained from Brodmann area 19 in response to the flash stimulus. **(D)** Correlation analysis of data obtained from Brodmann area 17 in response to the pattern stimulus. **(E)** Correlation analysis of data obtained from Brodmann area 18 in response to the pattern stimulus. **(F)** Correlation analysis of data obtained from Brodmann area 19 in response to the pattern stimulus. HC, healthy control; FP, form perception; NFP, no form perception; VC, visual cortex; SPL, superior parietal lobule; ITG, inferior temporal gyrus; FEF, frontal eye field. Black dots: HC subjects, blue dots: FP patients, and red dots: NFP patients.

Figure 6 shows the functional and structural correlations of Brodmann areas 17, 18, and 19 in the flash and pattern visual tasks. Spearman’s rank correlation coefficients between the response intensity and GMV were examined in flash and pattern visual tasks. In the flash visual task, the response intensity showed a significant positive correlation with the GMVs of Brodmann area 17 (*r* = 0.714, *P* < 0.001, Figure 6A), Brodmann area 18 (*r* = 0.733, *P* < 0.001, Figure 6B), and Brodmann area 19 (*r* = 0.509, *P* = 0.003, Figure 6C). For the pattern stimulus, the response intensity also showed a significant positive correlation with the GMVs of Brodmann area 17 (*r* = 0.721, *P* < 0.001, Figure 6D), Brodmann area 18 (*r* = 0.728, *P* < 0.001, Figure 6E), and Brodmann area 19 (*r* = 0.363, *P* = 0.038, Figure 6F).

## Discussion

This study was the first to objectively and quantitatively evaluate visual function in patients with advanced RP using MRI. We found that visual task-related fMRI was a feasible method to objectively and quantitatively discriminate FP and NFP patients with advanced RP. The response intensity of the VC showed a significant difference between patients with FP and NFP during pattern visual tasks, and FC among ROIs also showed a significant difference between patients with FP and NFP in the flash visual task. The structure of the VC was altered in patients with advanced RP, and the degree of the structural deficit was positively correlated with the response intensity in the two visual tasks.

The treatment of advanced RP is still a challenge for clinical ophthalmologists. Some new therapeutic methods, such as gene therapy (Ducloyer et al., 2020), stem cell therapy (He et al., 2014), optogenetic therapy (Tomita and Sugano, 2021), and visual prosthesis (Castaldi et al., 2016), are being studied. An objective examination of visual function is an essential requirement for advanced RP therapy because the therapeutic effect should be evaluated objectively and quantitatively. However, an objective examination of visual function in patients with advanced RP is difficult to achieve with traditional clinical examinations used in ophthalmology, such as BCVA, VF, or visual electrophysiology. Furthermore, an ophthalmologist could only evaluate visual function based on the patient’s subjective report, which might indicate aspects such as LP, HM, and figure counting. As shown in our previous study, full-field electroretinogram, pattern electroretinogram, and pattern visual evoked potential cannot be used to evaluate the visual function of patients with advanced RP at all, but flash visual evoked potential might be useful to differentiate between patients with FP and NFP. This finding indicated differences in the degree of visual function of the VC in patients with advanced RP. However, flash visual evoked potential results in only a two-dimensional curve that includes limited information. Therefore, we used fMRI to evaluate the visual function of patients with advanced RP and found that it is a potential method to objectively and quantitatively detect the residual visual function of patients with advanced RP (Zhang et al., 2021). In this study, we suggested that fMRI was a feasible method to objectively and quantitatively discriminate FP and NFP patients with advanced RP.

An fMRI study has shown that severe impairment of RP had fewer V1 BOLD responses in contrast sensitivity visual tasks (Castaldi et al., 2019), another finding that was consistent with our study. However, due to the limited number of subjects, the study did not report a difference in different levels of patients with advanced RP, whereas our study showed that the response intensity of participants in the NFP group was significantly lower than that of participants in the FP group. A resting-state study revealed that FC in patients with RP was lower than that in normal controls and that neural activity synchronicity in the VC was reduced in patients with RP (Dan et al., 2019). Our study also revealed that under visual task conditions, the FC in patients with RP was lower than that in normal controls. Moreover, the FC of part of the pathway in participants in the NFP group was significantly lower than that of participants in the FP group. A previous study observed a significantly decreased GMV of V1 (Brodmann area 17) in patients with RP, and this decreased GMV was related to the degree of VF loss (Rita Machado et al., 2017). In our study, the result was similar, but we found that the GMVs of Brodmann areas 18 and A19 were also decreased. In addition, the average GMVs of Brodmann areas 17, 18, and 19 were positively correlated with the average response intensity of these areas in both flash and pattern visual tasks, providing the structural basis of VC functional deficits in patients with RP.

In the visual task, we found that the pattern visual task evoked greater differences in the response intensity of VC between participants in the FP group and NFP group than the flash visual task, but only the flash visual task evoked a difference in ipsi_V1–contra_V1 FC between participants in the FP group and NFP group. Moreover, the flash visual task elicited greater differences in the visual network between the RP group and HC group. This result was due to the different characteristics of the two visual tasks and the deficit exhibited by patients with RP. The flash visual task entailed white light stimulation, and participants in both the FP and NFP groups were able to detect the light signal; thus, the response intensity of the VC was not very different. However, the stimulation in the pattern visual task employed a white-black checkerboard design, participants in the FP group were able to reasonably detect more of a visual signal in the pattern task, and a greater difference was observed between the participants in the FP and NFP groups in this task than in the flash task.

The visual function deficit of RP starts in the peripheral retina and progresses to the central retina (Phelan and Bok, 2000; Shintani et al., 2009). The dorsal stream of the visual system receives the visual signal from the surrounding part of the retina, and the ventral stream receives the visual signal from the center of the retina (Mahon et al., 2007; Laycock et al., 2011). Therefore, the dorsal stream was damaged more substantially in patients with RP in the early stages, and the ventral stream was damaged in these patients later. In the FC comparison, the flash visual task mainly evoked the dorsal visual stream activation because it contains high contrast and rapid visual information. Therefore, the visual network showed a greater deficit in the flash task. The pattern visual task provided shape and detailed visual information, and thus, it mainly activated the ventral visual stream. Therefore, the visual network did not show obvious deficits in the pattern visual task. Moreover, the simple shape and detailed visual signal of the pattern visual task would mainly be processed in the VC and did not evoke obvious activation in the visual network.

In patients with advanced RP, poor visual function results in poor fixation and eye movement function. Eye movements might also evoke cortical responses. In addition, fMRI signals revealed this response; however, eye movement related to the cortical area mainly includes the FEF and the SPL (Hanes et al., 1998; Shikata et al., 2008). In this study, the ROI with significantly different activity was the VC, but not the FEF and SPL. Therefore, the activity in the VC was mainly evoked by visual task stimulation but not eye movements.

Despite the limited number of patients with RP included in this study, considerably significant differences were observed. A limitation of this study was that the visual function of the patients with RP was divided into two levels (FP and NFP). Due to the limited number of patients with RP presenting equal levels of vision in both eyes, we were unable to set additional levels of visual function. If we can recruit more patients with RP with equal levels of vision in both eyes in the future, a more precise and objective evaluation of visual function could be performed.

## Conclusion

Functional magnetic resonance imaging was an effective tool to objectively and quantitatively differentiate patients with RP presenting FP and NFP. The pattern visual task had a greater ability than the flash visual task to differentiate patients with RP into FP and NFP groups according to their response intensity in the VC, but the flash visual task displayed a greater ability to differentiate FC in the ipsi_V1–contra_V1 pathway between patients with RP from the FP and NFP groups. The GMVs of both patients with RP from the FP and NFP groups decreased, and the degree of the decrease was positively correlated with the VC response intensity in both flash and pattern visual tasks.

## Data availability statement

The raw data supporting the conclusions of this article will be made available by the authors, without undue reservation.

## Ethics statement

The studies involving human participants were reviewed and approved by the Human Ethics Committees of Southwest Hospital approved the protocol (2017 scientific research No. 17), and all subjects gave their written informed consent. The patients/participants provided their written informed consent to participate in this study.

## Author contributions

HW: research design and manuscript writing. WO: data collection and analysis. YL, JW, and ZY: research guiding. MZ: subjects collection. HZ: subjects collection. All authors contributed to the article and approved the submitted version.

## Footnotes

1
http://rfmri.org/dpabi

2
https://www.fil.ion.ucl.ac.uk/spm/

3
https://www.nitrc.org/projects/bnv

4
http://dbm.neuro.uni-jena.de/vbm8/

5
https://www.alivelearn.net/xjview/

6
http://www.restfmri.net
